# Cross-Sectional Analysis of Obesity and Serum Analytes in Males Identifies sRAGE as a Novel Biomarker Inversely Associated with Diverticulosis

**DOI:** 10.1371/journal.pone.0095232

**Published:** 2014-04-16

**Authors:** Sarah S. Comstock, Markita M. Lewis, Dorothy R. Pathak, Kari Hortos, Bruce Kovan, Jenifer I. Fenton

**Affiliations:** 1 Department of Food Science and Human Nutrition, Michigan State University, East Lansing, Michigan, United States of America; 2 Department of Epidemiology and Biostatistics, Michigan State University, East Lansing, Michigan, United States of America; 3 College of Osteopathic Medicine, Michigan State University, East Lansing, Michigan, United States of America; 4 Tri-County Gastroenterology, Professional Corporation, Clinton Township, Michigan, United States of America; Florida International University, United States of America

## Abstract

Diverticulosis can lead to diverticulitis, a colon condition involving inflammation and other complications. Diverticulosis can result from biological, behavioral, or genetic causes. However, the etiology of diverticulosis is unknown. Although diet is associated with diverticulosis, recent studies suggest other factors influence risk. We sought to identify anthropometric or serum markers that were associated with the presence of diverticulosis. To determine these associations, 126 asymptomatic men (48–65 yr) were recruited at the time of preventative screening colonoscopy. Anthropometric measures were taken, and blood was collected for serum protein analysis. Data were analyzed by logistic regression and factor analysis. Obese individuals (BMI >30) were 7.8 (CI: 2.3–26.3) times more likely than normal weight (BMI <25) individuals to have diverticulosis. The relationship was similar for waist circumference. Individuals with a waist circumference >45 inches were 8.1 (CI: 2.8–23.8) times more likely to have diverticulosis than those with a waist circumference <38 inches. Leptin was also positively associated with diverticulosis (OR = 5.5, CI: 2.0–14.7). Both low molecular weight adiponectin (LMW, OR = 0.50; CI: 0.3–0.8) and the soluble receptor for advanced glycation end products (sRAGE, OR = 0.4, CI: 0.3–0.7) were inversely related to the presence of diverticulosis. sRAGE levels were not correlated with leptin or C-peptide concentrations. The pattern of high BMI, waist circumference, leptin and C-peptide increased the odds of diverticulosis while the pattern of high levels of sRAGE and LMW adiponectin decreased the odds of diverticulosis. Associations between diverticulosis and anthropometric or serum markers may elucidate the origins of diverticulosis and may enable physicians to identify individuals at risk for diverticulitis.

## Introduction

Diverticulosis affects approximately half of Americans over the age of 60, and two-thirds by 80 years of age, but most individuals who have diverticulosis are unaware of their status until complications arise [1]. Diverticulosis is characterized by small pouches that form within weak spots in the lining of the colon and bulge outward. These pouches can consequently become inflamed. The inflamed condition is called diverticulitis. When inflammation does occur, complications such as bleeding, infections, or small tears within the colon often follow [2]. Complications can lead to colectomy or other surgical procedures, resulting in significant medical expenses. Diverticular disease has direct costs of approximately $3.5 billion each year in the United States, making it one of the most expensive digestive diseases to diagnose and treat [3], [4].

Diverticulosis can result from biological, behavioral, or genetic causes. Some previously reported risk factors of diverticulosis include aging, smoking, lack of dietary fiber, obesity, and hereditary disease [1], [5], [6]. However, recent research has called into question the role of dietary fiber and highlighted the potential role of BMI in diverticular disease [5]. BMI has also been previously correlated with diverticulitis [7]. Obesity increases concentrations of inflammatory molecules in the blood [8], [9]. These molecules, also known as adipokines and cytokines, subsequently circulate through the body promoting inflammation and negatively affecting organs such as the heart or intestine [10]–[12]. Thus, although several possibilities exist, the exact etiology of diverticulosis is currently unknown.

Tumor necrosis factor (TNF)-α is the only inflammatory molecule associated with diverticulosis and diverticular disease to date [13], [14]. The relationships between diverticulosis and factors such as other serum cytokines, serum adipokines, or serum markers of insulinemia are poorly characterized. Identification of anthropometric or serum markers indicative of diverticulosis risk may provide mechanistic clues to the etiology of diverticular diseases and potential targets for prevention or treatment. Therefore, the purpose of this study was to identify anthropometric and/or serum markers that are associated with the presence of diverticulosis.

## Materials and Methods

### Ethics Statement

Consent forms and all materials associated with this cross-sectional study were approved by the institutional review board of Michigan State University on January 12, 2009 (IRB#08-786). At the time of enrollment, immediately prior to sample and data collection, written informed consent was obtained from each participant.

### Study Population

Between August 2009 and February 2011, healthy males ranging from 48–65 years of age were recruited from either the Tri-County Gastroenterology Clinic (Clinton Township, MI) or the Michigan State University Clinic (East Lansing, MI) at the time of colonoscopy screening. Participants were undergoing screening colonoscopies and were asymptomatic. Exclusion criteria included: 1) cancer within the past two years, 2) surgery within the past two years (including colon surgery), 3) inflammatory bowel diseases (e.g., Crohn's, ulcerative colitis), 4) autoimmune disorders (e.g., Rheumatoid arthritis, HIV/AIDS, Lupus), 5) diabetes, 6) chronic liver or kidney disease, 7) history of heart failure, 8) current immunosuppressant usage, 9) asthma, chronic obstructive pulmonary disease or other lung problems, 10) familial adenomatous polyposis, and 11) Lynch syndrome or hereditary non-polyposis colorectal cancer. 126 men (>96% white) participated in the study. At the time of enrollment, immediately prior to colonoscopy, written informed consent was obtained and clinical metadata on subject co-morbidities, current medications, and family history were collected. Also at the time of enrollment, venous blood was drawn and serum was isolated by standard procedures and stored at −80°C. Full colonoscopy was performed on each participant. During the colonoscopy, a gastroenterologist (MSU-affiliated clinics, MI) categorized the segment of the colon in which diverticula or polyps were found. Specimens collected during colonoscopy were sent to regional medical center Pathology Departments for histopathological analysis by board-certified pathologists.

### Anthropometry

At the time of enrollment, anthropometric measures were taken to calculate BMI and to record waist circumference. Trained staff measured participants' height, weight, and waist circumference following standard procedures. The staff attended two training sessions and demonstrated acceptable inter-observer reliability for all measures. All measurements were conducted in duplicate, and the average of two measures was used for analysis. Scales and stadiometers were calibrated with standard weights and heights prior to the first measurement of the day. Height was measured to the closest 0.1 cm using a wall-mounted stadiometer (SECA 214; Seca Corp, British Indicators Ltd., Birmingham, UK). Body weight was measured to the closest 0.2 kg on a digital platform scale accurate to 200 kg (BWB-800AS Digital Scale, Tanita, Arlington Heights, LA). Body weight was measured in light clothing with empty pockets and without shoes, watches, and other accessories. BMI was calculated as weight (kg) divided by height squared (m^2^). Weight status was defined as normal weight (BMI <25), overweight (25≤BMI<30) and obese (BMI≥30).

### Serum Analyte Analysis

Multimeric adiponectin (total, high (HMW), medium (MMW) and low (LMW) molecular weight) was measured by enzyme-linked immunosorbent assay (ELISA) following the manufacturer's instructions (Alpco Diagnostics, Salem, NH). Human leptin ELISA kits were used according to the manufacturer's instructions (R&D Systems, Minneapolis, MN). C-peptide concentrations were determined using a C-peptide ELISA (Calbiotech, Spring Valley, CA) as directed by manufacturer's protocol. The Synergy HT plate reader (Bio-Tek, Winooski, VT) was used to measure ELISA absorbencies. Serum concentrations of IL-6, IL-1α, IL-1β, IL-10, and TNF-α, were determined using a MILLIPLEX™ MAP Human Cytokine/Chemokine panel (HCYTOMAG-60K, Millipore, Billerica, MA) as per the manufacturer's protocol. The soluble receptor for IL-6 (sIL-6R) and the soluble receptor for advanced glycation end products (sRAGE) were analyzed using a MILLIPLEX™ MAP Human Soluble Cytokine Receptor panel (HSCR-32K, Millipore). MILLIPLEX™ samples were analyzed on a Bio-Plex 100 using Bio-Plex 4.1 software (Bio-Rad, Hercules, CA).

### Statistical Analysis

Sample size was arrived at by power calculations based on data obtained from the first 80 enrolled subjects. Frequencies, means, standard deviations and ranges were calculated. Correlation among variables was determined using Pearson correlation. Categorical variables were constructed using either biological cut off points (BMI, presence of diverticulosis) or tertiles within the data (leptin, adiponectin, LMW, MMW, HMW, IL-6, IL-1α, IL-1β, IL-10, TNF-α, sIL-6R). BMI was set to three categories: < 25 (normal), ≥ 25 but < 30 (overweight), or ≥ 30 (obese). Presence of diverticulosis was coded as “no diverticulosis” or “diverticulosis present”.

Age was treated as a continuous variable. IL-6, IL-1α, IL-1β, IL-10, and TNF-α values were missing for 1 individual (0.8% missing-ness). Each individual was assigned a smoking status of “never smoked” (fewer than 100 cigarettes in lifetime) or “ever smoked”. Smoking status was missing for 22 individuals (17.5% missing-ness). All missing data were considered missing at random. Multiple imputation (seed  =  20121119, imputations  =  7) was used to impute the 1 value missing for IL-6, IL-1α, IL-1β, IL-10, TNF- α, and all missing smoking data [15].

Odds ratios (OR) were determined using polytomous logistic regression models for categorical outcome data with more than two levels. Otherwise OR were determined using logistic regression. All models were adjusted for age and smoking status because these factors have been previously reported to be associated with diverticulosis [5]. Test for trend was carried out across categories for the variables of interest. Because imputation was used, multiple imputation analyze (Proc MIANALYZE) was used to determine the results from analysis of the imputed data sets. SAS version 9.3 (SAS Institute Inc.; Cary, NC) was used for all statistical analysis. p ≤ 0.05 indicates significance.

Biomarker patterns were generated by factor analysis (Proc Factor, SAS). Factor analysis reduces the number of variables generating latent variables called factors. A linear transformation is then performed to enable interpretation. In this case, varimax rotation was performed. Three factors were retained as determined by eigenvalues >1. The procedure assigns each person a factor score for each of the three factors that emerged from the data. Logistic regression of tertiles for each factor score was used to determine the odds of having diverticulosis, using the first tertile as the reference group.

## Results

Of the 126 men enrolled in the study, 53 men (42%) had diverticulosis. 28 of those with diverticulosis also had at least one polyp, and of those with at least one polyp, 18 individuals had at least one adenoma. However, the presence of diverticulosis was not significantly associated with the presence of polyps or polyp type.

**Figure 1:**
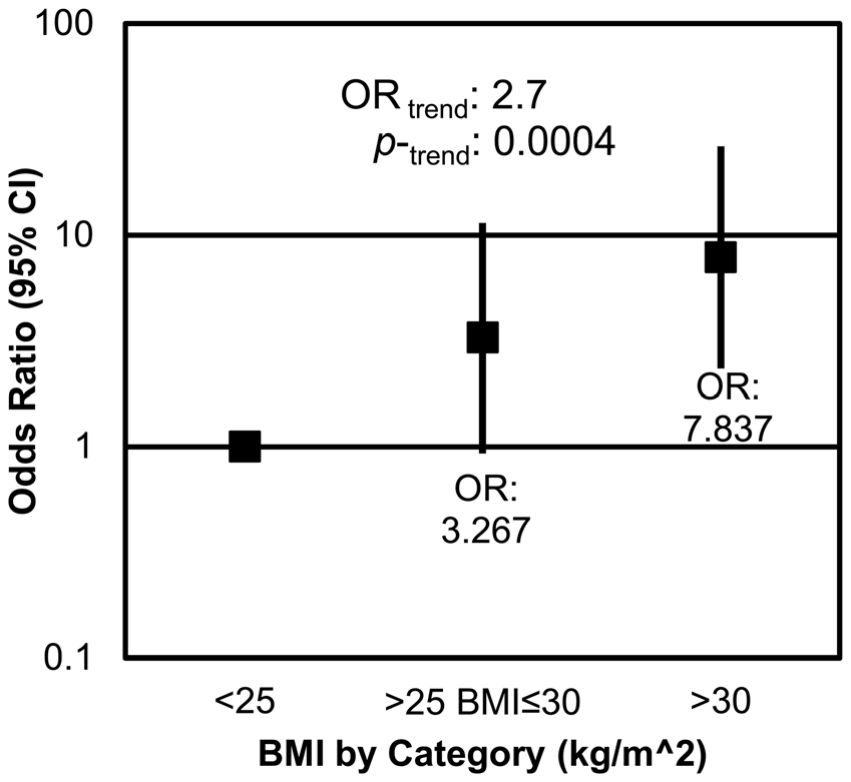
BMI is positively associated with presence of diverticulosis. Overweight individuals were 3.3 (CI: 0.9–11.3) times more likely, and obese individuals were 7.8 (CI: 2.3–26.3) times more likely to present with diverticulosis than normal weight individuals. With each increase in BMI category, the odds of having diverticulosis increased by 2.7 (CI: 1.6–4.7) fold.

**Figure 2:**
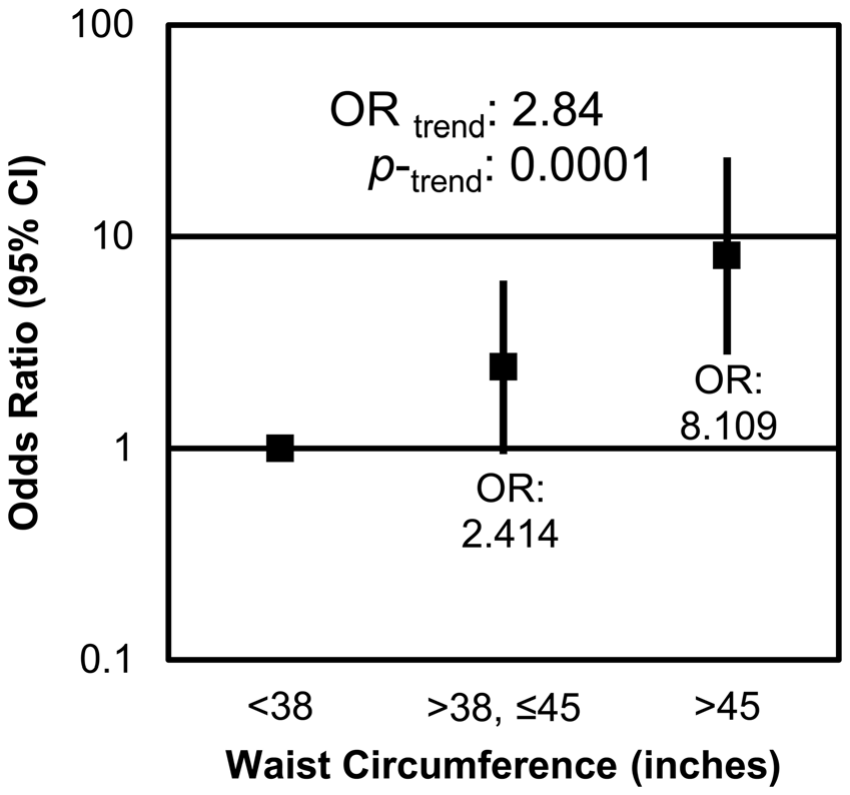
Waist circumference is positively associated with the presence of diverticulosis. Individuals with waist circumferences between 38–45 inches were 2.4 (CI: 0.9–6.2) times more likely to have diverticulosis than those with waist circumferences below 38 inches. Those with waist circumferences above 45 inches were 8.1 (CI: 2.8–23.8) times more likely to present with diverticulosis than individuals with waist circumferences below 38 inches. For each increase in waist circumference tertile, an individual's odds of having diverticulosis increased by 2.8 (CI: 1.7–4.9) fold.

Within the sample, 27 of the participants were normal weight, 47 were overweight, and 52 were obese. Among the participants who had diverticulosis, 4 were normal weight, 18 were overweight, and 31 were obese. Obesity was strongly associated with the presence of diverticulosis in measures of both BMI and waist circumference. Participants who were classified as obese (BMI >30) were significantly more likely to have diverticulosis than their normal weight (BMI<25) counterparts (p = 0.0009), with an odds ratio of 7.8 (CI: 2.3–26.3). Additionally as BMI increased, the odds of an individual having diverticulosis increased 2.7 (CI: 1.6–4.7) times (**Figure 1**). Waist circumference, an anthropometric measure correlated with obesity and, more specifically, visceral adiposity, was also shown to be positively associated with the presence of diverticulosis (**Figure 2**). Participants whose waist circumferences were between 38–45 inches tended to be 2.4 (CI: 0.9–6.2) times more likely to have diverticulosis than those who had waist circumferences below 38 inches (p = 0.07). Additionally, participants with waist circumferences above 45 inches were 8.1 (CI: 2.8–23.8) times more likely to have diverticulosis than those with waist circumference smaller than 38 inches (p = 0.0001).

**Figure 3:**
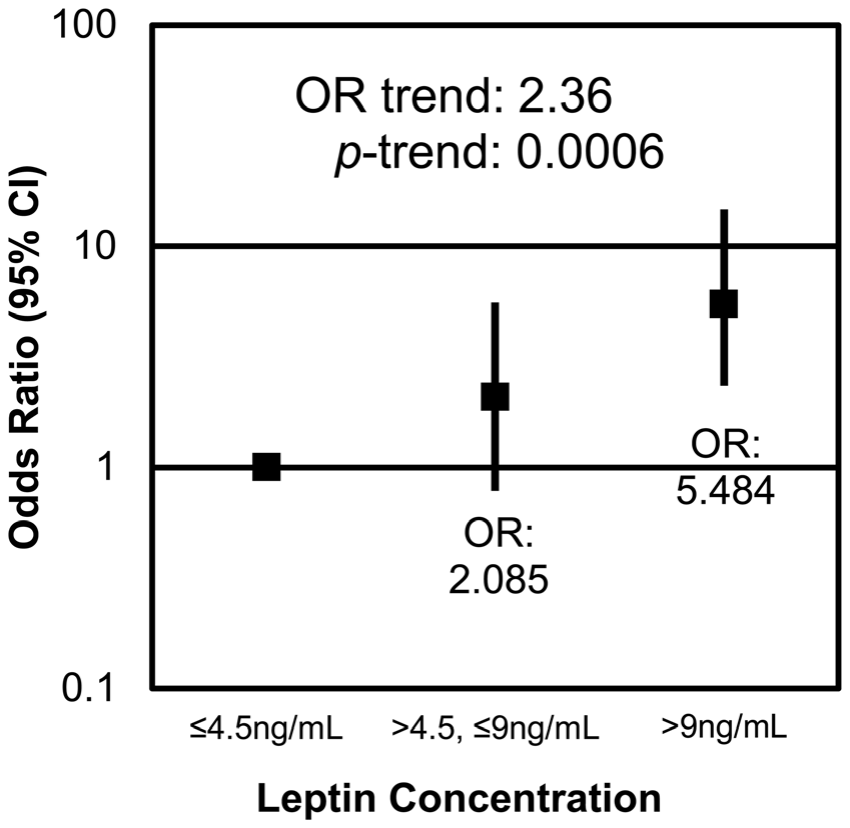
Leptin is positively associated with the presence of diverticulosis. Individuals who had serum leptin concentrations between 4.5 and 9 ng/ml were 2.1 (CI: 0.8–5.6) times more likely and those with concentrations above 9 ng/ml were 5.5 (CI: 2.0–14.7) times more likely to have diverticulosis than individuals with serum concentrations below 4.5 ng/ml. For each tertile increase in serum leptin concentration the risk of having diverticulosis increased 2.4 (CI: 1.4–3.9) times.

**Figure 4:**
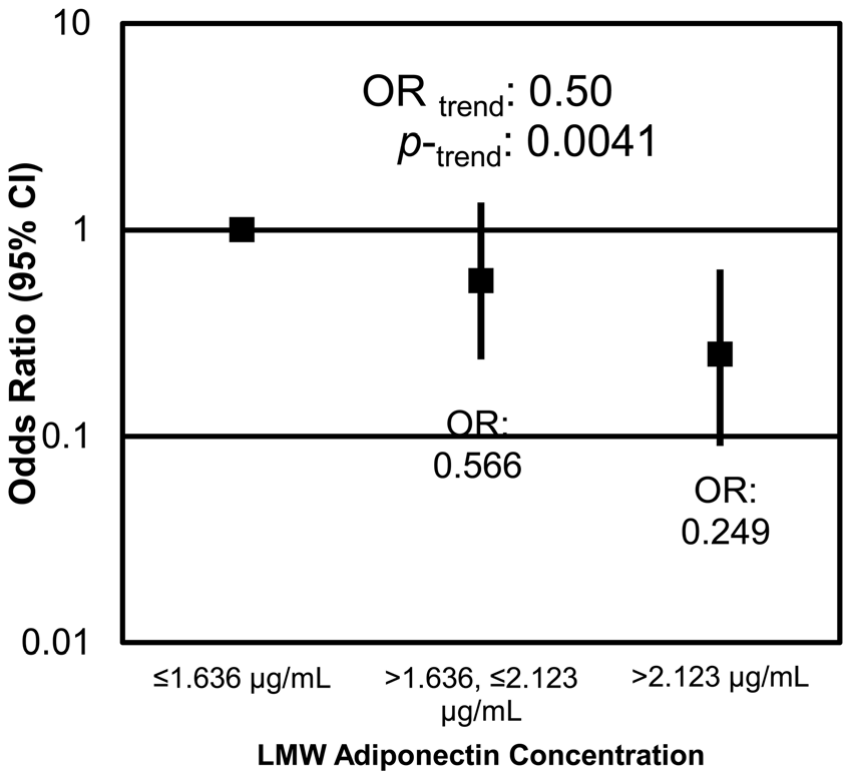
LMW adiponectin is inversely associated with the presence of diverticulosis. Individuals with serum LMW adiponectin concentrations between 1.6–2.1 µg/mL were 0.6 (CI: 0.2–1.4) times less likely to have diverticulosis, and those with concentrations above 2.1 µg/mL were 0.2 (CI: 0.09–0.6) times less likely to have diverticulosis than individuals with concentrations below 1.6 µg/mL. With each tertile increase of LMW adiponectin, the odds of having diverticulosis decreased 0.5 (CI: 0.3–0.8) fold.

The adipokines leptin and low molecular weight (LMW) adiponectin were significantly associated with the presence of diverticulosis. Males who exhibited the highest concentrations of leptin (>9 ng/ml) were 5.5 (CI: 2.0–14.7) times more likely to have diverticulosis than those who had concentrations of leptin below 4.5 ng/ml (p = 0.0007) (**Figure 3**). An inverse relationship existed between LMW adiponectin and the presence of diverticulosis. With each tertile increase in LMW adiponectin, the odds of having diverticulosis decreased by half (OR = 0.5, CI: 0.3–0.8; p = 0.0041) (**Figure 4**). Individuals with serum LMW above 2.1 µg/ml were 0.2 times less likely than those with serum LMW below 1.6 µg/ml to have diverticulosis (p = 0.0041).

**Figure 5:**
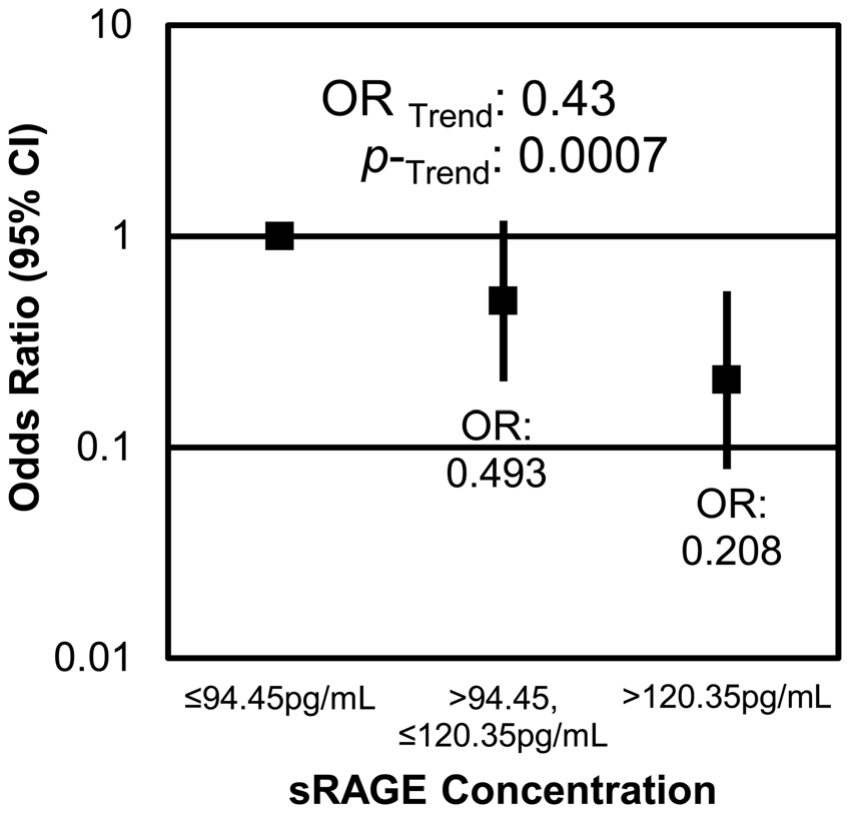
sRAGE is inversely related to the presence of diverticulosis. Males with serum sRAGE concentrations between 94.5 and 120.4/ml were 0.5 (CI: 0.2–1.2) less likely to have diverticulosis, and males with sRAGE concentrations above 120.4 pg/ml were 0.2 (CI: 0.08–0.5) times less likely to have diverticulosis than those with sRAGE concentrations below 94.5 pg/ml. For each tertile increase in serum sRAGE, the odds of having diverticulosis decreased by 0.4 (CI: 0.3–0.7) times.

Another serum analyte that had a strong association with diverticulosis was sRAGE. sRAGE was inversely associated with the presence of diverticulosis. Participants who had serum concentrations of sRAGE in the highest tertile (>120.4 pg/mL) were less likely to have diverticulosis (OR = 0.2, CI: 0.08–0.5) than those who had the lowest concentration of sRAGE (p = 0.0014). Trend analysis showed that the odds of having diverticulosis decreased by 0.4 (CI: 0.3–0.7) with each tertile increase in serum sRAGE (p = 0.0014) (**Figure 5**). Serum sRAGE concentrations were negatively correlated with BMI and waist circumference, and positively correlated with serum concentrations of IL-6, IL-1α, IL-1β and TNF-α (**Table 1**). Serum sRAGE was not correlated with serum leptin or adiponectin concentrations.

**Table 1:** Pearson correlation coefficients for anthropometric measurements and serum analytes.

	C-peptide	sRAGE	sIL-6R	IL-1α	IL-1β	IL-10
BMI	**0.4805**	**−0.1836**	0.0551	0.1577	0.1355	0.0941
	**<0.0001**	**0.0396**	0.5402	0.0790	0.1319	0.2967
Waist Circumference	**0.5064**	**−0.2414**	0.0583	0.0730	0.0694	0.0129
	**<0.0001**	**0.0065**	0.5167	0.4186	0.4421	0.8864
TNF-α	**0.3166**	**0.2933**	0.1231	**0.6020**	**0.5824**	**0.2619**
	**0.0003**	**0.0009**	0.1713	**<0.0001**	**<0.0001**	**<0.0032**
C-Peptide		−0.0434	0.1146	0.1548	0.0937	0.1034
		0.6294	0.2012	0.0848	0.2986	0.2513
sRAGE	−0.0434		−0.0059	**0.2799**	**0.2571**	0.1463
	0.6294		0.9480	**0.0016**	**0.0038**	0.1034
Leptin	**0.6700**	−0.0449	0.1017	0.1336	0.0963	0.0593
	**<0.0001**	0.6173	0.2570	0.1375	0.2854	0.5114
Total Adiponectin	**−0.3091**	0.0306	−0.0223	−0.1109	−0.0842	−0.1543
	**0.0004**	0.7342	0.8046	0.2181	0.3505	0.0857
HMW Adiponectin	**−0.2820**	0.0093	−0.0061	−0.1303	−0.0945	−0.1473
	**0.0014**	0.9176	0.9461	0.1475	0.2944	0.1011
MMW Adiponectin	−0.1549	0.0084	0.0261	−0.0350	−0.0831	−0.1229
	0.0833	0.9256	0.7722	0.6981	0.3567	0.1721
LMW Adiponectin	**−0.2712**	0.0664	−0.0658	−0.0502	−0.0171	−0.0979
	**0.0021**	0.4603	0.4643	0.5782	0.8495	0.2776
IL-6	0.1636	**0.2570**	0.1707	**0.9518**	**0.7440**	**0.5651**
	0.0683	**0.0038**	0.0570	**<0.0001**	**<0.0001**	**<0.0001**
sIL-6R				0.1514	0.1595	0.1419
				0.0919	0.0757	0.1145
IL-1α					**0.7491**	**0.5231**
					**<0.0001**	**<0.0001**
IL-1β						**0.8168**
						**<0.0001**

Note: The top number is the Pearson correlation coefficient. The P values are listed under the correlation coefficient.

C-peptide, an indicator of hyperinsulinemia, was not significantly associated with the presence of diverticulosis. However, individuals with C-peptide levels above 3.3 ng/ml tended to be more likely to have diverticulosis than individuals with C-peptide levels below 1.8 ng/ml (p = 0.097). Serum concentrations of C-peptide were positively correlated with BMI, waist circumference, leptin and TNF-α, and negatively correlated with total, HMW and LMW adiponectin (**Table 1**). C-peptide concentrations were not correlated with serum levels of the other inflammatory cytokines measured, including IL-6, IL-1α, and IL-1β.

The cytokines analyzed in our study (IL-6, sIL-6R, IL-1α, IL-1β, IL-10, and TNF-α) were not found to be associated with the presence of diverticulosis. With the exception of TNF-α, none of these cytokines were correlated with BMI or waist circumference (**Table 1**, [16]). In addition, although serum LMW adiponectin was negatively associated with the presence of diverticulosis, total adiponectin, and the other isoforms of adiponectin (MMW and HMW) were not associated with the presence of diverticulosis (**Table 2**).

**Table 2:** Association of serum analytes with diverticulosis.

		Trend
		OR
	OR (95% CI)	(p trend)
MMW adiponectin (µg/mL)		
≤0.5	1	
>0.5 to ≤ 0.8	1.27 (0.51–3.14)	1.37
>0.8	1.87 (0.75–4.68)	(0.1784)
HMW adiponectin (µg/mL)		
≤1.2	1	
>1.2 to ≤ 2.4	1.01 (0.42–2.43)	0.77
>2.4	0.60 (0.25–1.48)	(0.2728)
Total Adiponectin (µg/mL)		
≤3.6	1	
>3.6 to ≤ 5.3	1.29 (0.53–3.15)	0.79
>5.3	0.62 (0.25–1.52)	(0.2908)
TNF-α (pg/mL)		
≤5.9	1	
>5.9 to ≤ 8.6	1.39 (0.56–3.44)	1.24
>8.6	1.53 (0.63–3.72)	(0.3492)
IL-6 (pg/mL)		
≤0.3	1	
>0.3 to ≤ 6.0	1.12 (0.45–2.78)	1.24
>6.0	1.54 (0.64–3.73)	(0.3353)
IL-1β (pg/mL)		
≤0.03	1	
>0.03 to ≤ 3.3	0.52 (0.21–1.32)	1.17
>3.3	1.40 (0.56–3.46)	(0.5006)
IL-1α (pg/mL)		
≤3.9	1	
>3.9 to ≤ 18.1	0.43 (0.17–1.08)	1.04
>18.1	1.09 (0.45–2.66)	(0.8574)
IL-10 (pg/mL)		
≤3.1	1	
>3.1 to ≤ 12.3	1.06 (0.43–2.61)	1.23
>12.3	1.51 (0.61–3.71)	(0.3714)
sIL-6R (ng/mL)		
≤18.4	1	
>18.4 to ≤ 25.2	1.46 (0.60–3.58)	1.07
>25.2	1.15 (0.47–2.82)	(0.7628)
C-peptide (ng/mL)		
≤1.8	1	
>1.8 to ≤ 3.3	1.10 (0.44–2.75)	1.47
>3.3	2.15 (0.87–5.32)	(0.0940)

Note: Models were adjusted for age and ever/never smoked.

As a next step, factor analysis was used to predict how combinations of these variables might be predictive of diverticulosis. The factor loading matrix is shown in **Table 3**. The factor loadings are correlations between each biomarker and each factor. Three factors were identified, factor 1: pro-inflammatory biomarkers, factor 2: obesity-associated biomarkers and factor 3: protective biomarkers. Factors 2 and 3 were significantly associated with the presence of diverticulosis (**Table 4**). Although factor 1 was not associated with a change in odds of diverticulosis (OR: 1.3, CI: 0.8–2.0, p = 0.31), a high factor 2 score was associated with an increased odds for diverticulosis (OR: 2.8, CI: 1.7–4.6, p<0.0001), while a high factor 3 score was associated with a decreased odds for diverticulosis (OR: 0.5, CI: 0.3–0.8, p = 0.0063).

**Table 3:** Factor loading matrix for biomarkers of diverticulosis in asymptomatic adult males.

	Factor 1	Factor 2	Factor 3
**Waist circumference**	0.02^1^	**0.91**	−0.18
**BMI**	0.11	**0.89**	−0.13
**Leptin**	0.01	**0.89**	0.18
**C-peptide**	0.05	**0.74**	0.21
**IL-1β**	**0.93**	0.04	0.11
**IL-6**	**0.88**	0.09	0.24
**IL-1α**	**0.86**	0.10	0.29
**IL-10**	**0.84**	−0.002	−0.17
**TNF-α**	**0.52**	0.34	0.54
**sRAGE**	0.17	−0.18	**0.79**
**LMW**	−0.10	−0.50	**0.25**

1 The factor loading value indicates the correlation between the variable and the factor.

**Table 4:** Association of biomarker patterns with diverticulosis.

		Trend
		OR
	OR (95% CI)	(p trend)
Factor 1:		
Pro-Inflammatory		
Tertile 1	1	
Tertile 2	2.45 (1.00–5.98)	1.26
Tertile 3	1.52 (0.62–3.73)	(0.3051)
Factor 2:		
Obesity-Associated		
Tertile 1	1	
Tertile 2	3.19 (1.19–8.52)	2.77
Tertile 3	6.91 (2.57–18.59)	(<0.0001)
Factor 3:		
Protective		
Tertile 1	1	
Tertile 2	0.51 (0.21–1.22)	0.52
Tertile 3	0.27 (0.11–0.67)	(0.0063)

## Discussion

In this study we analyzed the association between diverticulosis and anthropometric or serum markers. Several were found to be significant. High BMI and large waist circumference were associated with greater odds of presenting with diverticulosis. sRAGE, a highly novel marker, which was not correlated with leptin or adiponectin but was correlated with BMI and waist circumference, was inversely related to the presence of diverticulosis. LMW adiponectin, which was inversely correlated with BMI and waist circumference, was inversely related to the presence of diverticulosis. Furthermore, leptin, which positively correlated with BMI and waist circumference, was positively associated with the odds of having diverticulosis. Other serum analytes were not significantly associated with the presence of diverticulosis.

Other studies have also found an association between obesity and diverticular disease [17]–[20]. An early prospective study analyzing the relationship between BMI, waist circumference, waist-to-hip ratio, and diverticulitis and/or diverticular bleeding demonstrated an increased risk among obese individuals [21]. Many studies, when observing obesity and diverticular disease, use solely BMI as an anthropometric measure. In addition, most rely on self-report of body weight, height and waist circumference. However, trained staff took anthropometry measurements in our study, and waist circumference was included in our analysis. The inclusion of waist circumference as a factor is important because waist circumference is highly correlated with central adiposity and strongly associated with health risk indicators for metabolic syndrome [22], [23]. By using a measure based on visceral adiposity, such as waist circumference, we could detect relationships between local adipose and diverticulosis. In addition the use of BMI enabled us to detect relationships between total body mass and diverticulosis.

Obese individuals experience constant inflammation mediated through the production of adipokines. Leptin and adiponectin are two major proteins secreted by the adipose tissue [24]. Leptin plays a role in the direct regulation of immune cells and adipocytes. It plays a role in the responsiveness and proliferation of regulatory T cells and the production of inflammatory cytokines such as TNF-α, IL-1α, IL-1β, IL-6, and IL-4 [25]. Leptin concentrations are increased in animals with intestinal inflammation and humans with inflammatory bowel diseases. Adiponectin is generally considered an anti-inflammatory protein produced by the adipose tissue. Circulating levels of adiponectin decrease as adipose within the body increases [26]. Adiponectin circulates throughout the body in three forms—LMW, MMW, and HMW. There may be functional differences between the multimers of the protein. HMW adiponectin may have pro-inflammatory effects, increasing the production of cytokines such as IL-6 and TNF-α. However, LMW adiponectin has been associated with decreased IL-6, and negatively correlated with waist circumference, meaning that its increased concentrations are inversely related to adiposity and obesity [27]. Therefore the strong positive association between leptin and diverticulosis, and the strong negative association between LMW and diverticulosis may be a result of their strong correlation with anthropomorphic measures. However, it may be that these adipose-associated molecules directly contribute to diverticulosis via other mechanisms.

Because obesity is associated with low grade inflammation, and the selected cytokines had been previously identified as inflammatory biomarkers associated with other inflammatory bowel diseases [28], we expected to find strong associations of inflammatory molecules with diverticulosis. However, there were no significant associations of the measured serum cytokines (IL-6, IL-1α, IL-1β, IL-10, and TNF-α) with the presence of diverticulosis. Surprisingly, TNF-α was not positively associated with the presence (p = 0.3) of diverticulosis in our study. Several studies by Tursi et al. report that TNF-α increased with the severity of diverticular disease [13], [14]. However, our subjects were asymptomatic and undergoing preventive screening colonoscopy, so the severity of diverticular disease and the TNF-α concentrations among the participants were lower than those reported in the Tursi studies.

Caution must be used when interpreting the results of this study as there are some limitations. Because this study was conducted in primarily white, adult males, the results may not apply to individuals of other ethnicities or females. Although, individuals with diagnosed pre-diabetes/diabetes or taking medications related to glucose dysregulation were excluded from the study, it is possible some participants had hyperglycemia and/or hyperinsulinemia. Our smoking classification of “ever” or “never” does not reflect the extent of smoking. Thus, these factors may confound our findings. The associations described in this study were based on cross-sectional data therefore the factors associated with diverticulosis may not cause diverticulosis. Traditionally, diverticular disease has been classified into stages based on the findings of Hinchey et al. in 1978 or a modified version of the Hinchey classification [29]. Because the participants in this study had asymptomatic diverticulosis, which is the lowest severity on most diverticular disease severity scales, the associations reported herein may not be found in individuals with more serious disease. Finally, the associations identified will need to be confirmed in other populations, and the ability of these factors to predict the presence of diverticulosis will need to be established in future studies.

We have identified biomarker patterns which can either increase the odds of diverticulosis (factor 2: obesity-associated biomarkers) or decrease the odds of diverticulosis (factor 3: protective biomarkers). A comparison of the factor loading matrix between the present population and the factor loading matrix(matrices) from a future research population(s) would determine whether the biomarker patterns identified in this study allow for the suitable prediction of diverticulosis. Such future studies will need to be conducted in populations with similar participant characteristics as ours. However, once validated, these patterns could be tested for their suitability to predict diverticulosis in broader populations.

This study is unique because it describes associations between serum analytes and diverticulosis that have not been analyzed previously. These analytes included sRAGE, IL-10, IL-1α, IL-1β, total adiponectin and its isoforms as well as sIL-6R. Importantly, we have identified a novel biomarker (sRAGE) that may also be mechanistically involved in the process by which obesity influences diverticulosis. Advanced glycation end products (AGEs) result from non-enzymatic reactions between sugars and free amino groups on proteins. The receptor for AGEs (RAGE) is a cell surface protein, and the interaction between AGEs and RAGE leads to an inflammatory state [30], [31]. The soluble receptor for advanced glycation end products, sRAGE, is found in serum and competes with its membrane-bound counterpart, RAGE, to bind AGEs [32]. When serum concentrations of sRAGE are high, it prevents inflammation by inhibiting the interaction between AGEs and cell surface RAGE. sRAGE is decreased in some chronic inflammatory conditions such as chronic pulmonary obstructive disease (COPD), chronic kidney disease, and Type II diabetes [32]. In a prospective case-cohort study, Finnish male smokers within the highest quintile of sRAGE concentration had a lower relative risk for colorectal cancer than those within the lowest quintile of sRAGE (RR = 0.65; 95% CI, 0.39–1.07) [33]. Thus, the inverse relationship between sRAGE and diverticulosis found in our study is consistent with studies relating serum levels of sRAGE to intestinal disease states. Importantly, this is the first study demonstrating that sRAGE is inversely associated with diverticulosis.

Although serum sRAGE was inversely correlated with BMI and waist circumference, it was not correlated with the adipokines leptin or adiponectin. This lack of correlation with the adipokines suggests a role for sRAGE in diverticulosis independent of the leptin/adiponectin signaling axis. sRAGE was positively correlated with serum inflammatory cytokines including IL-6, IL-1α, IL-1β, and TNF-α. IL-6, IL-1α, and IL-1β were not correlated with BMI or waist circumference. In addition, sRAGE was not correlated with C-peptide, a marker of hyperinsulinemia. Furthermore, with the exception of TNF-α, C-peptide was not correlated with the inflammatory cytokines. Therefore a unique inflammatory signature was associated with high serum sRAGE concentrations. The identification of this unique signature and its association with diverticulosis may provide novel insight into the signaling pathways involved in diverticulosis within the gastrointestinal tract. We hypothesize that the increased levels of sRAGE in our subjects are an effective response to chronic low-grade inflammation. Rising levels of serum sRAGE, would, in turn, inhibit the inflammatory response that typically causes complications in diverticular disease. Thus, when sRAGE is present and the inflammatory response is inhibited fewer complications would arise.

Future research should determine mechanisms by which serum sRAGE concentrations may influence diverticulosis, and how combinations of sRAGE and inflammatory cytokines might be used to predict progression to diverticulitis. The identification of the pathway by which sRAGE interacts with intestinal epithelial cell health would enable drug development or other interventions to be designed for the prevention of diverticulosis or other intestinal diseases. In addition, because high concentrations of sRAGE may prevent progression to or complications of diverticular disease in individuals with diverticulosis, future research should address strategies to increase serum concentrations of sRAGE. For instance, physical activity can increase sRAGE concentrations in patients with Type 2 diabetes [34]. Physical activity could also decrease BMI and other anthropometric measures associated with diverticulosis in our study. Thus, increased physical activity may be one potential intervention strategy to prevent diverticular disease. Vitamin D supplementation is another intervention that could potentially affect the progression of diverticular disease through modulation of serum sRAGE concentrations. Increased levels of serum calcitriol levels were associated with increased sRAGE concentrations [35].

About 4–15% of individuals with diverticulosis will progress to diverticular disease [36]. The identification of anthropometric (BMI and waist circumference) and serum analytes (sRAGE, leptin, LMW adiponectin) associated with diverticulosis may yield insight into the etiology of diverticular disease. In addition, the factors identified might be molecular targets for the prevention of diverticular disease. Prevention of progression to diverticulitis would significantly decrease medical spending on this ailment that commonly afflicts elderly adults.
